# Supplementary material for: Anti-Cancer Effects of Oxygen-Atom-Modified Derivatives of Wasabi Components on Human Leukemia Cells
Source: Int J Mol Sci. 2023 Apr 6;24(7):6823. doi: 10.3390/ijms24076823 (PMC10095376; doi:10.3390/ijms24076823)
Supplement: Supplementary file 1 [file ijms-24-06823-s001.zip › ijms-2318712-supplementary.pdf]

S1:

A. p-Histone H3

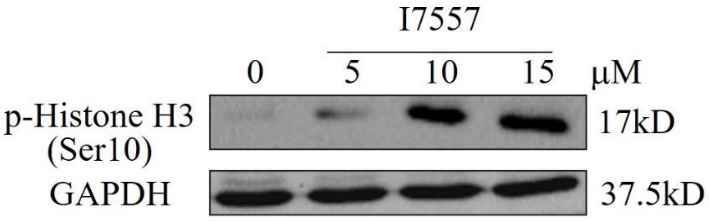

B. p-Chk-1

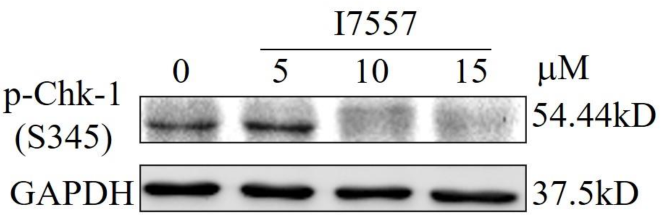

C. p-Chk-2

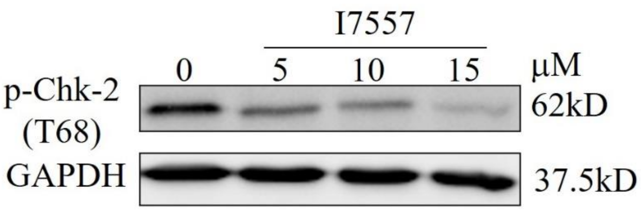

D. p-Cdc25c

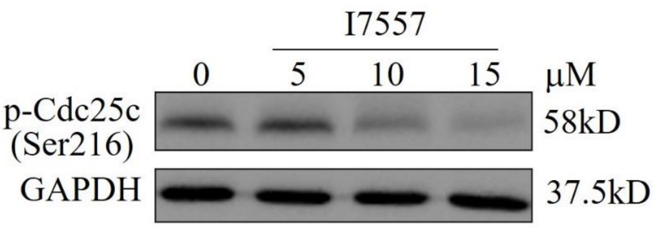

E. p-Cdc2

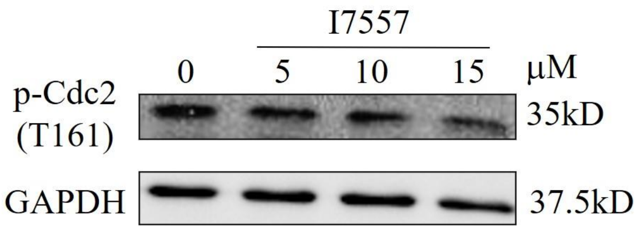

F. Cyclin-B1

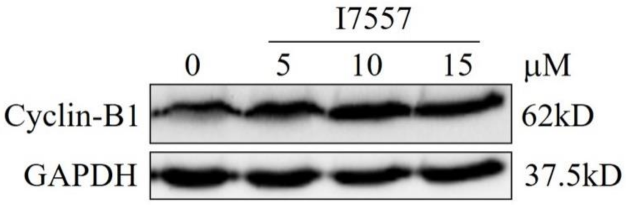

G. p-Plk-1

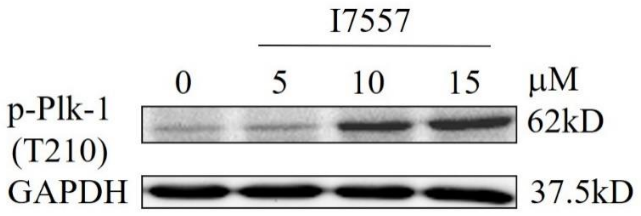

Supplementary Figure S1:
The expression of cell cycle related proteins in K562 cells. Cells were treated by I7557 (0 – 15 μM) for 48 hours.

S2:

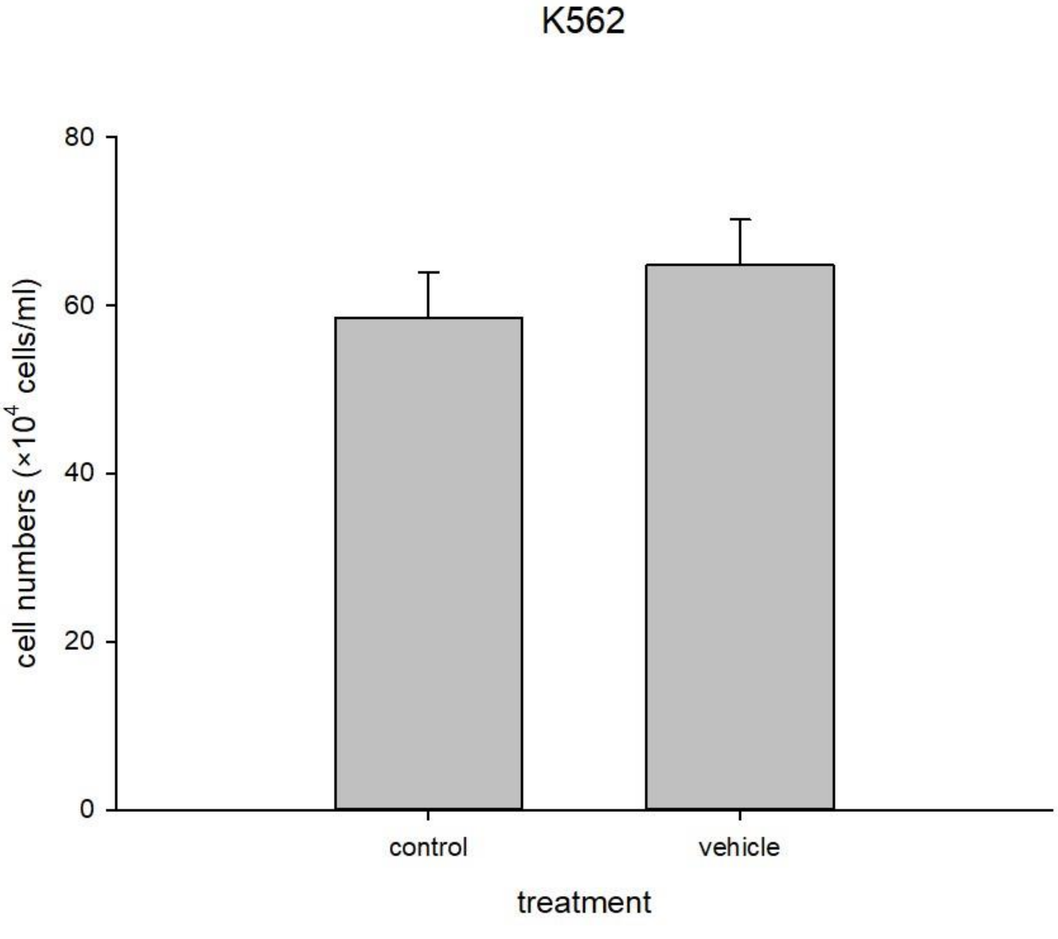

Supplementary Figure S2:
There is no significant difference of cell viability with comparison of control group and vehicle group (DMSO).
